# Main Protease From SARS‐CoV‐2 Dysregulates Glucose Handling in the C2C12 Cell Line In Vitro: A Mechanistic Study

**DOI:** 10.1002/iid3.70383

**Published:** 2026-02-27

**Authors:** Praise Tatenda Nhau, Mlindeli Gamede, Andile Khathi, Ntethelelo Sibiya

**Affiliations:** ^1^ Pharmacology Division, Faculty of Pharmacy Rhodes University Makhanda South Africa; ^2^ Physiology, University of Pretoria Tshwane South Africa; ^3^ Human Physiology, University of KwaZulu‐Natal Durban South Africa

**Keywords:** diabetes mellitus, GLUT 4, insulin, insulin resistance, Mpro, SARS‐COV‐2

## Abstract

**Background:**

There is evidence demonstrating the risk of developing diabetes mellitus because of SARS‐CoV‐2 infection. Therefore, further research is needed to determine pathological mechanisms at which SARS‐CoV‐2 induces diabetes mellitus. This study therefore aims to understand the effect of SARS‐CoV‐2 Main protease (M^pro^) on glucose uptake and GLUT‐4 translocation as well as AKT, GLUT‐4, and IL‐6 expression in skeletal muscle (C2C12).

**Methods:**

In this study, C2C12 cell preparations were exposed to different concentrations of M^pro^ (2.5, 5, 10, 20, 40, 80, and 160 nmol/mL) for 24 h to evaluate cytotoxicity and glucose uptake. For further assays, only the higher concentrations (40, 80, and 160 nmol/mL) were used. The impact of M^pro^ on cell viability, glucose uptake, AKT, GLUT‐4 and IL‐6 expression, GLUT‐4 translocation as well as lipid peroxidation were analyzed.

**Results:**

Following 24 h of treatment with SARS‐CoV‐2 M^pro^, C2C12 cells were viable. The baseline and insulin‐stimulated glucose uptake were impaired in the C2C12 cell line. M^pro^ also compromised GLUT4 translocation and expression in the C2C12 cell line compared to the control. Baseline and Insulin‐stimulated AKT were not significantly altered in the presence of M^pro^. Intracellular and extracellular IL‐6 levels were also affected by M^pro^. An increase in MDA levels, a marker for lipid peroxidation, was observed.

**Conclusions:**

The observations suggest that the SARS‐CoV‐2 M^pro^ may be inducing an insulin‐resistant state and dysregulation of glucose metabolism. Further studies are warranted to fully elucidate the mechanisms underlying the development of new‐onset diabetes mellitus in patients with a history of COVID‐19.

## 1 Introduction

Diabetes mellitus (DM) is a well‐established risk factor for poor clinical outcomes in patients with COVID‐19 [1]. However, emerging evidence suggests a bidirectional relationship between the two conditions [1]. Among the repercussions of the COVID‐19 pandemic were significant socioeconomic disruptions, such as increased strain on healthcare systems due to an increase in non‐communicable diseases such as DM [2]. DM encompasses a group of chronic metabolic disorders marked by impaired glycemic control, resulting in elevated blood glucose concentrations due to either insulin resistance or insufficient insulin production [3]. DM is classified into several categories, with the two primary types being Type 1 DM (T1DM), characterized by lack of insulin secretion, and Type 2 DM (T2DM), which is marked by insulin resistance [4]. The other types of diabetes include gestational diabetes, maturity onset diabetes of the young, neonatal diabetes, and latent autoimmune diabetes in adults [5]. The prevalence of DM is high in developing countries like South Africa, leading to both morbidity and mortality [6]. On a global scale, the prevalence of DM is expected to rise from 415 million individuals in 2015 to 642 million by 2040, highlighting a substantial increase in the burden of the disease [7]. Sedentary lifestyles and unhealthy diet habits, including consuming refined carbohydrates, may be contributing factors to the rise in DM cases [8]. With the global rise in DM, it is essential to investigate the underlying pathophysiological mechanisms contributing to increase in the DM prevalence.

A significant number of individuals may have undiagnosed prediabetes, a condition that is characterized by impaired fasting glucose and/or impaired glucose tolerance that is frequently linked with low‐level inflammation [9]. Research conducted in the past 30 years has demonstrated that, in addition to pre‐diabetes and DM, obesity can also contribute to low‐grade inflammation, which increases the risk of systemic insulin resistance and the development of DM [3]. Individuals with pre‐diabetes and insulin resistance due to obesity can often maintain normal glucose tolerance for several years before transitioning abruptly to T2DM [10, 11]. Some researchers have suggested that viral infections may play a role in triggering this transition from pre‐diabetes to T2DM [3, 11].

COVID‐19 has been linked to disruptions in glucose regulation, with hospitalized patients frequently exhibiting hyperglycemia [12, 13, 14]. However, the precise mechanisms underlying SARS‐CoV‐2‐induced glycemic disturbances remain incompletely understood, and a clear consensus has yet to be established. However, research has established a link between DM and increased susceptibility to SARS‐CoV‐2, as well as a higher risk of severe COVID‐19 complications. Conversely, COVID‐19 itself has been implicated in the onset of DM, although it remains uncertain whether the virus triggers T1DM, T2DM, or an atypical form of diabetes distinct from the conventional classifications [15, 16]. During the 2003 SARS‐CoV‐1 outbreak described by Pal and Banerjee et al. as a “cousin” of SARS‐CoV‐2, a study on 39 individuals without a prior history of DM was conducted and it was found that 20 developed the condition during hospitalization [16]. Despite glycemic management, two patients had persistent DM after a 3‐year follow‐up. Given that this glycemic disruption resulted from a less virulent and less transmissible SARS‐CoV‐1, the impact of SARS‐CoV‐2 is likely to be even more significant. This study is therefore justified by the urgent need to address the potential long‐term health impacts of COVID‐19, particularly those with pre‐existing comorbidities such as DM. With the rising prevalence of DM even before the pandemic, understanding how SARS‐CoV‐2 exacerbates conditions like chronic inflammation, metabolic stress, and insulin resistance is crucial for predicting and mitigating future public health challenges. Given the complex interactions between SARS‐CoV‐2 and metabolic pathways, understanding how the virus impacts glucose metabolism and insulin sensitivity is of paramount importance. The importance of this study also lies in its potential to uncover new insights into the molecular interactions between SARS‐CoV‐2 and glucose metabolism. Identifying these pathways could lead to the development of more targeted therapies aimed at preventing or managing DM in individuals affected by COVID‐19. Additionally, these findings would fill a critical gap, inform public health strategies, providing a foundation for more effective and tailored interventions to address the rising global incidence of DM, especially in the wake of the pandemic. By integrating COVID‐19‐related metabolic risks into broader healthcare strategies, this research could help reduce the long‐term burden of DM on individuals and healthcare systems alike. The findings could offer insights into targeted interventions to mitigate the risk of DM in post‐COVID‐19 populations, advancing both clinical care and public health strategies [17].

This in vitro study therefore aimed to elucidate the mechanisms associated with SARS‐CoV‐2 in key insulin‐sensitive cell lines. This study mainly focused on the SARS‐CoV‐2 protein, main protease (M^pro^) and its effect on the insulin signaling pathway, inflammatory and oxidative stress markers. The chymotrypsin‐like cysteine protease (also known as main protease, Mpro or 3CLpro) of SARS‐CoV‐2 plays a vital role in allowing the virus to reproduce within host cells and is therefore essential for the replication of the virus itself [18]. According to literature, Mpro is approximately 34.21 kDa per monomer [19]. The enzyme is a homodimer with three domains: domain I (residues 8–101), domain II (residues 102–184), and domain III (residues 201–306) in each monomer [19]. The hydrolytic activity of 3CLpro is dependent on the formation of 3CLpro dimers. A cleft exists between domain I and domain II where the active site can be found [20]. A chymotrypsin‐like fold resembling the picornavirus 3C proteinase is found at the active site of SARS‐CoV‐2 Mpro, which is composed of the catalytic dyad Cys145 and His41 [21].

The envisaged impact of this research lies in its potential to uncover specific pathways through which SARS‐CoV‐2 could utilize to induce insulin resistance or impair glucose metabolism. Furthermore, these findings could provide a foundation for integrating COVID‐19‐related metabolic risks into broader public health strategies, addressing the rising incidence of DM with more informed, precise interventions.

## 2 Materials and Methods

### 2.1 Mpro and Insulin Preparation

Covid‐19 M^pro^ (CAE0172‐200UG) was obtained from Sigma‐Aldrich (St. Louis, USA) as a 0.2 mg powder. The stock solution (5900 nmol/mL) was prepared by dissolving M^pro^ in 1 mL DMSO, with working concentrations (2.5–160 nmol/mL) prepared using the dilution formula (*C*_1_ *V*_1_ = *C*_2_ *V*_2_). The solution was filtered (0.22 µm filter) and stored at −20°C. All preparations followed strict aseptic techniques, including the use of a biosafety cabinet, sterile consumables, and personal protective equipment to prevent contamination. A NovoRapid insulin vial (100 units/mL) was utilized. Before use, the vial was gently shaken for homogeneity. A stock solution (0.05 units/mL) was prepared by adding 5 µL of insulin to 10 mL DMEM in a sterile container, followed by thorough mixing with a vortex mixer. The solution was filtered (0.22 µm filter) and stored at 2°C–8°C. For experimental purposes, the working concentrations of the proteins were freshly prepared.

### 2.2 Cell Culture and Skeletal Muscle Differentiation

The assays were performed using C2C12 skeletal muscle cells, which were cultured in high‐glucose DMEM supplemented with 10% FBS and 1% penicillin–streptomycin. Cells were maintained at 37°C in a humidified incubator with 5% CO₂. Once they reached approximately 80% confluence, they were trypsinized and transferred to new tissue culture flasks before being seeded into 24‐ or 96‐well plates. C2C12 myoblasts underwent differentiation to promote myotube formation. Cells were seeded in the 24‐ or 96‐well plates depending on the assay to be conducted and maintained at 37°C in a 5% CO₂ atmosphere using high‐glucose DMEM. Once they reached 80% confluence, cells were rinsed with PBS (250 µL), and the growth medium was replaced with differentiation medium (DMEM with 2% FBS and 1% penicillin–streptomycin). The medium was refreshed daily for 4 days to support differentiation. Progress was monitored microscopically by observing elongated multinucleated cells.

### 2.3 Cell Viability Assay: MTT Assay

C2C12 were plated in the 96‐well plates at a density of (4.55 × 10^4^) cells/mL until they reached approximately 80% confluence. Cells were then treated with the different concentrations of M^pro^ (2.5,5, 10, 20, 40, 80, and 160 nmol/mL). Some wells containing cells were left untreated and contained media only to serve as the control group. The cells were incubated for 24 h at 37°C. After the treatment with the different concentrations of M^pro^, the culture medium was gently removed and the MTT solution (1 mg/mL) was added into each well. The plates were incubated for 3 h at 37°C in the dark. Thereafter, the MTT solution was discarded from each well followed by the addition of DMSO (100 μL/well) to dissolve the formazan crystals formed by the MTT solution. Subsequently, the cells were incubated for 10 min and the absorbance of the purple‐colored crystals was read using a spectrophotometer at a wavelength of 570 nm. The assay was performed in duplicate and each concentration was tested in sextuplicate. Cell viability was calculated as a percentage of the absorbance of the control group using the following equation:

$$\text{percentage cell viability}=\left(\frac{\text{average absorbance of treated wells}}{\text{average absorbance of control}}\right)\;\times 100.$$

### 2.4 Estimated Glucose Utilization Assay

The glucose utilization assay was carried out following the methodology outlined by Kuretu et al. with some modifications [22]. C2C12 seeded and placed into 24‐well plates, cultured and allowed to attach and obtain confluency, followed by experimentation.

After cell preparations were ready for experimentation, the old cell media was discarded, and 250 µL of media containing M^pro^ (2.5, 5, 10, 20, 40, 80, and 160 nmol/mL) was placed into the wells. A control group of cells was incubated with media only. The initial glucose readings were measured at time = 0 h. After an incubation time of 24 h, media glucose concentrations were recorded, again. To assess the effect of M^pro^ exposure on insulin sensitivity and its effect on the cell lines, the cells were pre‐incubated Mpro for 24 h followed by the treatment with media containing insulin (250 µL, 0.05 units/mL). A glucometer was used to read the media glucose measurements at both *t* = 0 and 24 h. Each concentration was tested in quadruplicate and the assay was repeated three times.

The estimation of glucose uptake was determined using the following equation:

$$\text{glucose uptake }(\%)=\left(\frac{\text{medium glucose}\left(T0\right)-\text{medium glucose}\;(T24)}{\text{medium glucose}\;(T0)}\right)\times 100.$$

### 2.5 In Cell ELISA: Semi‐Quantitative Expression of GLUT4, AKT, and IL‐6

All solutions were freshly prepared and used immediately. C2C12 cells were seeded in a 96‐well plate and cultured in DMEM for each ELISA experiment. After 24 h, the cells were treated with 100 μL of the different concentrations of M^pro^ (40, 80, and 160 nmol/mL) and incubated overnight. The following day, the media was aspirated and placed in vials for storage at 4°C. The control wells were set up to have a blank and negative control. The blank consisted of growth medium only and no cells and the M^pro^. The negative control consisted of cells that had the primary antibody and secondary antibody, but without the addition of M^pro^. While both controls aimed to measure non‐specific binding, the blank control assessed the background signal from the growth medium or any potential contaminants in the reagents used, without the influence of cells or M^pro^, while the negative control evaluated any potential background noise from the interaction of antibodies with cells in the absence of M^pro^.

For the in‐cell ELISA, paraformaldehyde (8%, 100 µL) solution was used to fix and cross‐link the cells to the microplate for 15 min at room temperature while shaking with a microplate shaker at 300 rpm. The paraformaldehyde was thereafter aspirated followed by a wash step, performed to eliminate any residual solution using 1× PBS (200 mL). After the final wash, the plates were blotted dry on a paper towel. The next step was the permeabilization of the cells using a permeabilization buffer (250 µL Triton X‐100 in 24.75 mL 1× PBS) for 30 min at room temperature on the plate shaker. After 30 min, the permeabilization buffer was aspirated from the wells and a blocking buffer (200 mL, prepared by adding 2 g of bovine serum albumin in 20 mL of 1× PBS) was added and incubated at room temperature for 2 h while shaking the plate at 300 rpm. After aspirating the blocking buffer, 100 µL of the primary antibody (AKT, GLUT4, and IL‐6) diluted at 1:5000 was added to each well separately. The plate was then incubated overnight at 4°C. Following incubation, the primary antibody was aspirated and the wells were washed three times with 250 µL of wash buffer (prepared by adding 625 µL Tween‐20 to 250 mL of 1× PBS) to remove excess and unbound antibody.

Afterwards, 100 µL of a secondary antibody (anti‐rabbit IgG, diluted 1:5000) specific to the primary antibody was added to each well. The plate was incubated at room temperature for 2 h with gentle shaking. After the incubation, the wells were washed four times with 250 µL of wash buffer, followed by the addition of 100 µL of TMB horseradish peroxidase substrate. The plate was incubated in the dark for 30 min. To stop the reaction, 100 µL of 0.1 M HCl was added, resulting in a color change from blue to yellow. The absorbance was then measured at 450 nm using a UV‐VIS spectrophotometer. The assay was performed in duplicates, with each concentration tested in triplicate. For GLUT4 translocation, the same procedure was used, except that the cell permeabilization step was omitted to detect only GLUT4 found across the membrane. The relative expression of each protein was calculated as follows:

$$\text{relative percentage expression}=\left(\frac{\text{average absorbance of treated wells}}{\text{average absorbance of control}}\right)\times 100.$$

### 2.6 Cell Culture Medium ELISA for IL‐6

The harvested media were used for extracellular assessment of IL‐6. The samples were centrifuged at a low speed of 300–500*g* and the supernatant was collected. A high‐binding 96‐well plate was prepared by adding 50 µL of DMEM and EMEM aspirated from the C2C12 cell line, followed by incubation at room temperature for 6 h. After the incubation period, the media was removed and the plate was blotted dry. No fixation or permeabilization steps were performed; however, the subsequent steps followed the same procedure as previously described in the in‐cell ELISA above. The percentage expression of each protein was also calculated using the previously mentioned formula.

### 2.7 Lipid Peroxidation: Media Malondialdehyde Concentration

Following cell treatment with Mpro, 250 µL of supernatant was aspirated and used for the TBARS assay. The harvested media was mixed with 400 µL of 2% phosphoric acid, where the resultant solution was further aliquoted into two separate tubes. Afterwards, phosphoric acid (200 μL of 7%) was added into both glass tubes followed by addition of HCl (400 µL, 3 mM) into one glass test tube (blank) and 400 μL thiobarbituric acid (TBA)/butylated hydroxytoluene (BHT) into the other test tube (sample test). To achieve an acidic pH of 1.5, 200 μL of 1 M HCl was added to both the blank and sample test tubes. All test tubes were boiled at 100°C for 15 min using the Labotec shaking Eco Bath water bath. They were then left to cool to room temperature for 10 min. After cooling, 1.5 mL of butanol was added to each test tube, which was then vortexed for 10 s. The test tubes were left to stand until the butanol phase (the top layer) became visible. Then, 200 mL of the butanol phase was added in triplicate to a 96‐well plate. The optical density of the samples was measured spectrophotometrically at 532 nm, with a reference wavelength of 600 nm, using the Varioskan Lux microplate reader. MDA concentrations (mM) were calculated by dividing the average absorbance of the samples by the absorption coefficient (156 Mmˉ^1^). The absorbance from these wavelengths was used to calculate the concentration of MDA using the Beer–Lambert law as follows:

$$\text{MDA concentration}=\left(\frac{\text{final absorbance}-\text{blank}}{\text{MDA coefficient}\;\left(156{\text{mmol}}^{-1}\right)\;}\right).$$

### 2.8 Statistical Analysis

Data are presented as mean ± SD from three replicates. Graphs were generated using GraphPad Prism (Version 8, GraphPad Software, San Diego, CA, Canada, 2019). Normality was assessed using normality and log‐normality tests in GraphPad Prism. If the data followed a normal distribution, one‐way ANOVA with Tukey's multiple comparison test was applied to compare M^pro^ concentrations with the control. For non‐normally distributed data, the Kruskal–Wallis test was used. An asterisk (*) denotes statistically significant differences (*p* ≤ 0.05) between the control and treatment groups.

## 3 Results

### 3.1 Cell Viability

**Figure 1:**
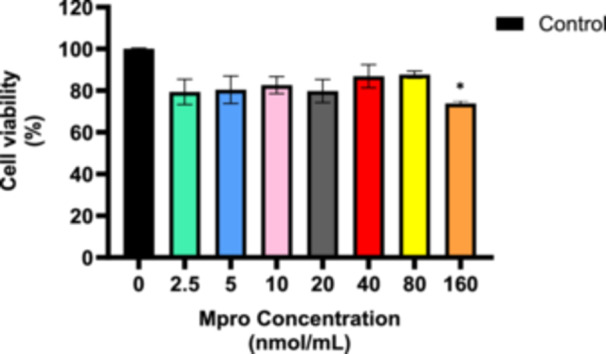
Cell viability was assessed after treating the C2C12 cell line with M^pro^ (2.5, 5, 10, 20, 40, 80, and 160 nmol/mL) for 24 h at different concentrations. Results are expressed as a percentage of viable cell count. Untreated cells were used as the control group. The values are expressed as mean ± standard deviation represented with error bars (*n* = 3). The asterisk (*) represents the statistical difference between the test compounds and the control at *p < 0.05.

A cytotoxicity study was conducted on the C2C12 cell line using various concentrations of 2. 5, 5, 10, 20, 40, 80, and 160 nmol/mL of M^pro^, as depicted in Figure 1. All concentrations generally demonstrated a decrease in cell viability by comparison to the control. There was no concentration‐dependent decrease observed; however, the highest concentration of M^pro^ showed a statistically significant decrease by comparison to the control. The highest concentration of 160 nmol/mL exhibited a decrease in cell viability, which was of statistical significance compared to the control.

### 3.2 Glucose Utilization

#### 3.2.1 Effect of M^pro^ in Non‐Insulin Stimulated Glucose Uptake

**Figure 2:**
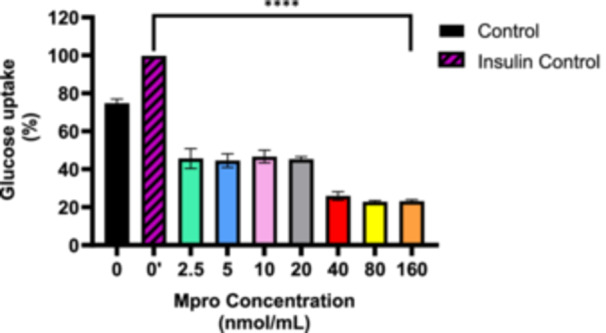
The relative glucose uptake was estimated after treating the C2C12 cell line with the different concentrations of M^pro^ (2.5, 5, 10, 20,40, 80, and 160 nmol/mL) for 24 h. Results are expressed as a percentage. Untreated cells were used as the control group. The values are expressed as mean ± standard deviation represented with error bars (*n* = 3). The asterisk (*) represents the statistical difference between the test compounds and the control at ****p < 0.0001.

The relative glucose uptake was measured after treating the C2C12 cell line with different concentrations of M^pro^ (2.5, 5, 10, 20, 40, 80, and 160 nmol/mL) for 24 h. The results are presented in Figure 2. The insulin‐treated control displayed higher glucose uptake than the untreated control, confirming that insulin effectively stimulated glucose transport, as expected. All M^pro^ concentrations showed a significant decrease in glucose uptake compared to the untreated control. M^pro^ treatments at increasing concentrations (from 2.5 to 160 nmol/mL) caused a progressive reduction in glucose uptake compared to the insulin‐treated and control groups. There is a dose‐dependent decrease in glucose uptake with an increase in M^pro^ concentration, with the most significant reduction observed at 160 nmol/mL.

#### 3.2.2 Insulin‐Stimulated Glucose Uptake in M^pro^‐Treated Cells

**Figure 3:**
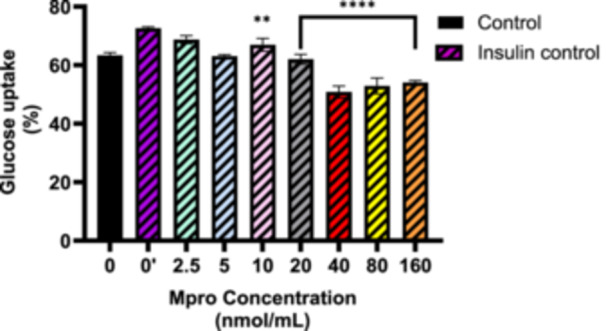
The relative glucose uptake was estimated after treating the C2C12 cell line with M^pro^ (2.5, 5, 10, 20, 40, 80, and 160 nmol/mL) for 24 h. Untreated cells were used as the control group. The values are expressed as mean ± standard deviation represented with error bars (*n* = 3). The asterisk (*) represents the statistical difference between the test compounds and the insulin control at **p < 0.01, and ****p < 0.0001.

The effect of M^pro^ on insulin‐stimulated glucose uptake was also performed using different concentrations of M^pro^ (2.5–160 nmol/mL). Figure 3 illustrates the effects of co‐administering insulin and M^pro^, with insulin serving as the reference control for statistical comparisons. The data show that insulin significantly enhances glucose uptake at lower M^pro^ concentrations compared to the control (untreated cells). However, as M^pro^ concentration increases, glucose uptake progressively declines. Insulin‐stimulated glucose uptake remained relatively high between 2.5 and 20 nmol/mL, though a slight but significant reduction is observed at 10 nmol/mL. The decline becomes more pronounced at higher concentrations, with the most significant reduction occurring at 160 nmol/mL. These findings suggest that while lower M^pro^ levels have minimal impact on insulin efficacy, higher concentrations may impair insulin‐stimulated glucose uptake.

#### 3.2.3 Relative GLUT4 Expression

**Figure 4:**
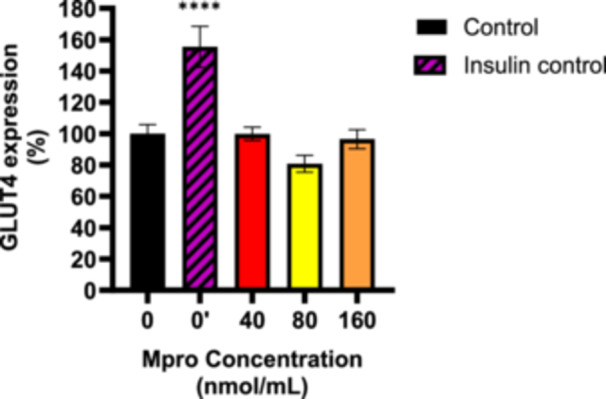
GLUT4 expression was estimated after treating the C2C12 cell with M^pro^ for 24 h at the different concentrations of 40, 80, and 160 nmol/mL. The values are expressed as mean ± SD represented with error bars (*n* = 3). Results are expressed as a percentage. Untreated cells were used as the control group. The asterisk (*) represents the statistical difference between the test compounds and the control at ****p < 0.0001.

**Figure 5:**
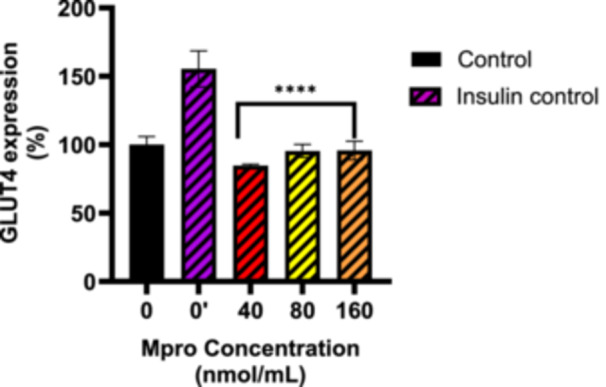
GLUT4 expression was estimated after treating the C2C12 cell with M^pro^ for 24 h at the different concentrations of 40, 80, and 160 nmol/mL. Following M^pro^ treatment, insulin was administered for 6 h. The values are expressed as mean ± SD represented with error bars (*n* = 3). Results are expressed as a percentage. The insulin group cells were used as the control group. The asterisk (*) represents the statistical difference between the test compounds and the control at ****p < 0.0001.

To investigate GLUT4 expression in C2C12 cells, we analyzed relative GLUT4 levels after M^pro^ treatment of concentrations of 40–160 nmol/mL under both baseline and insulin‐treated conditions. Figure 4 illustrates GLUT4 expression in C2C12 cells under baseline conditions (M^pro^ treatment only, with no insulin activation). The insulin treatment showed a significant increase in GLUT4 expression compared to the control (untreated) cells, indicating that insulin upregulates GLUT4 expression in skeletal muscle cells. For M^pro^ treatment, GLUT4 expression is comparable to the control (untreated), except for 80 nmol/mL where we observed a slight decrease in GLUT4 expression. No statistically significant difference was observed relative to the control (untreated cells). There was a statistically significant difference between insulin and the control (untreated cells). Figure 5 depicts GLUT4 expression in C2C12 cells following insulin treatment. Insulin treatment failed to significantly elevate GLUT4 expression levels across all M^pro^‐treated cells. Statistical analysis revealed significant differences in GLUT4 expression between the insulin‐treatment and M^pro^‐treated groups, ****p < 0.0001.

#### 3.2.4 Relative GLUT4 Translocation

**Figure 6:**
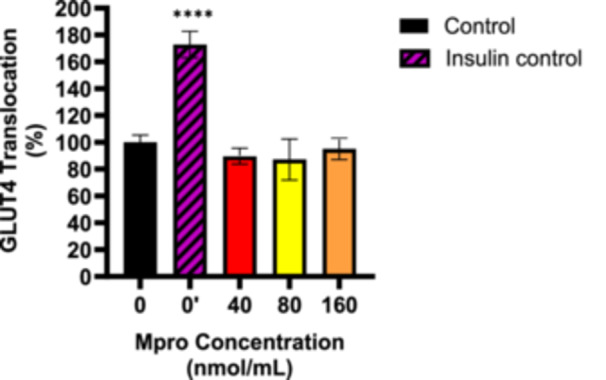
GLUT4 translocation was estimated after treating the C2C12 cell line with M^pro^ for 24 h at the different concentrations of 40, 80, and 160 nmol/mL. The values are expressed as mean ± SD represented with error bars (*n* = 3). Results are expressed as a percentage. Untreated cells were used as the control group. The asterisk (*) represents the statistical difference between the test compounds and the control at ****p < 0.0001.

**Figure 7:**
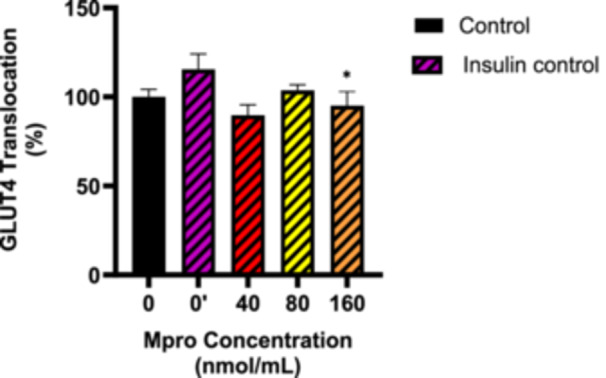
GLUT4 translocation was estimated after treating the C2C12 cell line with M^pro^ for 24 h at the different concentrations of 40, 80, and 160 nmol/mL. The values are expressed as mean ± SD represented with error bars (*n* = 3). Results are expressed as a percentage. Insulin‐treated cells were used as the control group. The asterisk (*) represents the statistical difference between the test compounds and the control at *p < 0.05.

The relative appearance of GLUT4 at the surface of the cell was assessed following treatment with M^pro^ at different concentrations of 40–160 nmol/mL as shown in Figure 6. There is a slight decrease in the appearance of GLUT4 for 40 and 80 nmol/mL M^pro^ treatment groups, followed by a slight increase for the 160 nmol/mL concentration compared to the control (untreated) cells. There was no statistically significant difference between the control (untreated cells) and all treatment conditions. To investigate the effect of M^pro^ on insulin‐stimulated GLUT4 translocation, the cells were pretreated with M^pro^ for 24 h after which insulin treatment followed for 5 h to activate GLUT4 translocation. Figure 7 displays the percentage of the GLUT4 that translocated to the plasma membrane for the C2C12 cell line. Insulin‐treated group (insulin only) showed a significant increase in GLUT4 translocation compared to the control (untreated) cells. Statistically significant decrease in GLUT4 translocation was observed between the M^pro^ treatment +insulin compared to the insulin‐treated group. Overall, insulin was unable to effectively allow for GLUT4 translocation in the presence of M^pro^ at all concentrations.

#### 3.2.5 Effect on AKT Expression

**Figure 8:**
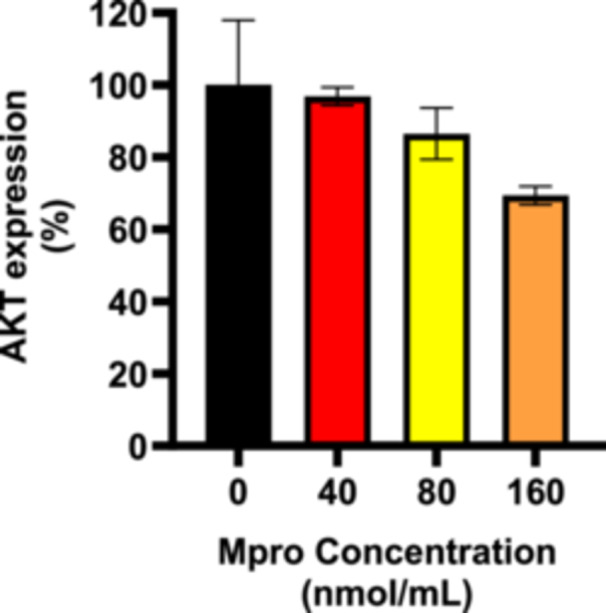
The relative expression of AKT in non‐insulin‐stimulated cells after treating C2C12 cell lines with M^pro^ (40, 80, and 160 nmol/mL). Values are expressed as mean ± SD represented with error bars (*n* = 3). Results are expressed as a percentage. Insulin‐treated cells were used as the control group.

**Figure 9:**
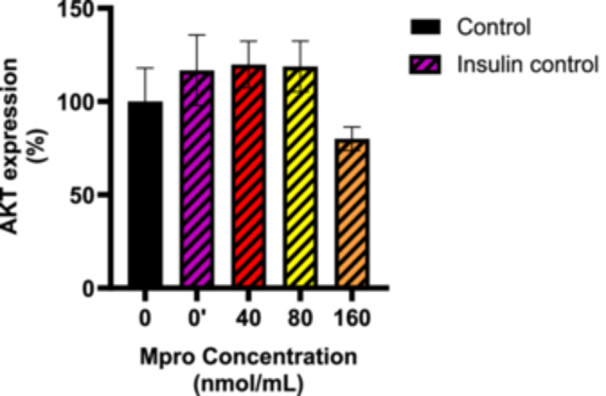
Effect of insulin‐stimulated AKT expression in M^pro^ pre‐treated cells. Values are expressed as mean ± SD represented with error bars (*n* = 3). Results are expressed as a percentage. Insulin‐treated cells were used as the control group.

To further assess any disturbances in the insulin signaling pathway, baseline expression of AKT and insulin‐stimulated production of AKT were analyzed in the C2C12 cell line after M^pro^ treatment at concentrations of 40–160 nmol/mL as illustrated in Figures 8 and 9, respectively. Figure 8 shows a steady decline in baseline AKT production levels, as the concentration of M^pro^ increased. The decrease suggests a dose‐dependent relationship, with the highest concentration of M^pro^ showing the most significant reduction in baseline AKT expression when compared to the control (untreated) cells. At 80 and 160 nmol/mL, there is a slight decline in AKT expression, though this change is not statistically significant. To assess the effect on AKT activation, we analyzed the relative expression of AKT in the C2C12 lines after co‐treatments of insulin and M^pro^, as shown in Figure 9. In the C2C12 cell line, the insulin control showed that there was a significant enhancement of AKT expression. Figure 9 shows that the insulin‐stimulated AKT levels remain relatively consistent across all concentrations of M^pro^ except for 160 nmol/mL when compared with insulin‐only control.

#### 3.2.6 Cellular and Medium IL‐6

**Figure 10:**
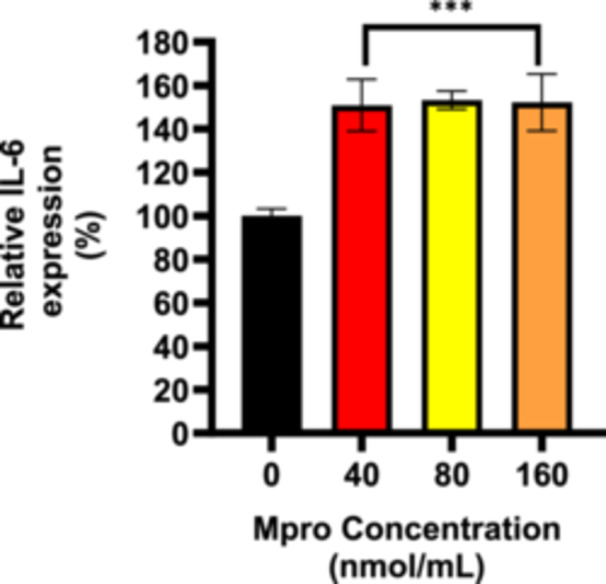
The relative cellular expression of IL‐6 was estimated after treating the C2C12 cell line with M^pro^ for 24 h at the different concentrations of 40, 80, and 160 nmol/mL respectively. The values are expressed as mean ± SD represented with error bars (*n* = 3). Results are expressed as a percentage. Untreated cells were used as the control group. The asterisk (*) represents the statistical difference between the test compounds and the control at ***p < 0.001.

**Figure 11:**
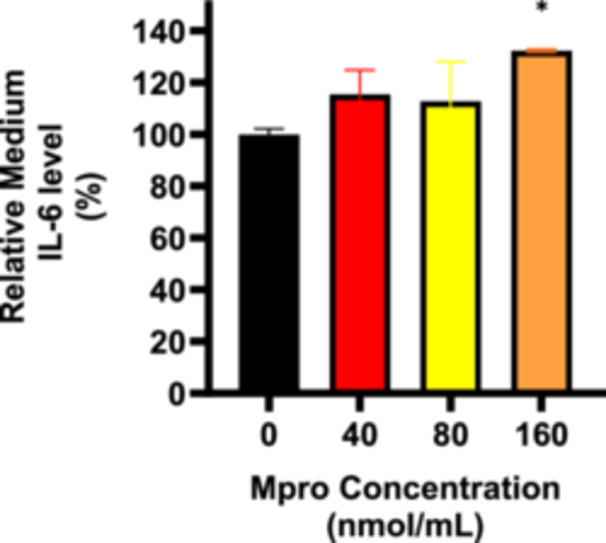
The relative IL‐6 concentration in cellular medium after treating the C2C12 cell line with M^pro^ for 24 h at the different concentrations of 40, 80, and 160 nmol/mL. The values are expressed as mean ± SD represented with error bars (*n* = 3). Results are expressed as a percentage. Untreated cells were used as the control group. The asterisk (*) represents the statistical difference between the test compounds and the control at *p < 0.05.

An intracellular ELISA was conducted to gain insight into IL‐6 expression within cells. Figure 10 illustrates the relative percentage in‐cell expression of IL‐6 in the C2C12 cells. A notable upregulation of IL‐6 expression was observed in the C2C12 cells for all the different treatment concentrations compared to the control (untreated) cells. Statistically significant differences were observed between the treatment and control groups, ***p < 0.001 suggesting that M^pro^ upregulates the expression of IL‐6 in C2C12 cells. Figure 11 depicts the relative percentage medium expression of IL‐6 in the C2C12 cells treated with M^pro^ at concentrations of 40, 80, and 160 nmol/mL, respectively. The results show an increase in the cellular expression of IL‐6 compared to the control (untreated) cells with the highest peak being for the highest concentration of M^pro^. No statistically significant difference was found between the control and treatment groups, except at the 160 nmol/mL concentration.

#### 3.2.7 Cellular and Medium MMP1

**Figure 12:**
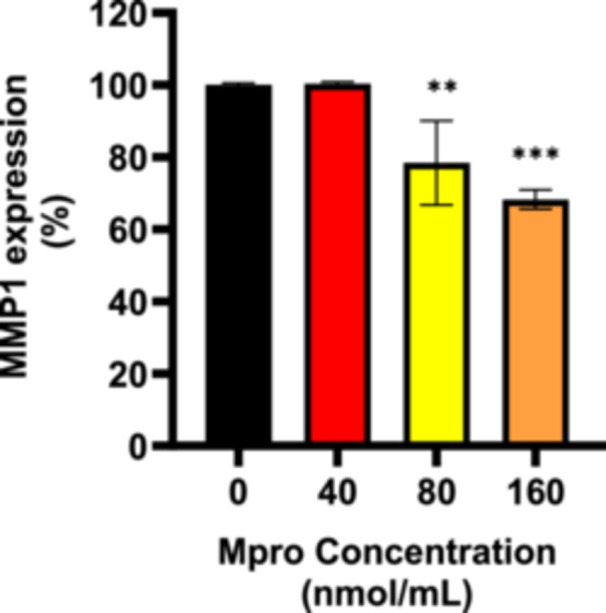
The relative expression of MMP1 was estimated after treating C2C12 cells with M^pro^ at the different concentrations of 40, 80, and 160 nmol/mL. The values are expressed as mean ± SD represented with error bars (*n* = 3). Results are expressed as a percentage. Untreated cells were used as the control group. The asterisk (*) represents the statistical difference between the test compounds and the control at **p < 0.01 and ***p < 0.001.

**Figure 13:**
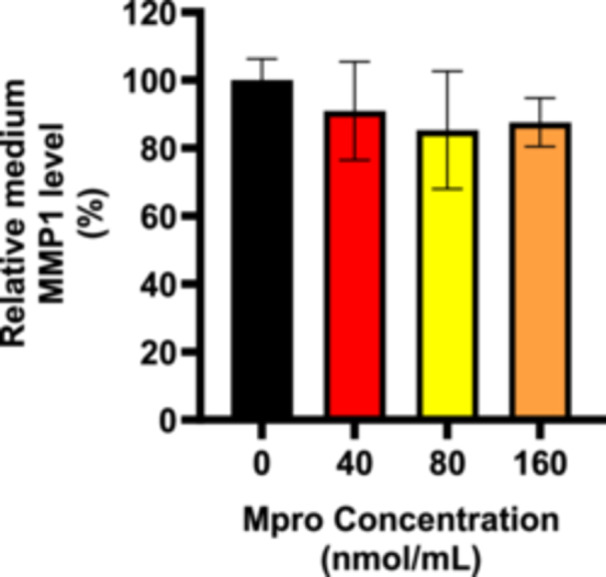
The relative concentration of medium MMP1 was estimated after treating C2C12 cells treated with M^pro^ for 24 h at the different concentrations of 40, 80, and 160 nmol/mL. The values are expressed as mean ± SD represented with error bars (*n* = 3). Results are expressed as a percentage. Untreated cells were used as the control group.

The relative expression of MMP1 was estimated after treating C2C12 cells with M^pro^ for 24 h at concentrations of 40, 80, and 160 nmol/mL as shown in Figure 12. There was a decrease in MMP1 expression with increasing concentrations of M^pro^. MMP1 expression remains close to the control level, showing a slight but non‐significant reduction for the lowest M^pro^ concentration, 40 nmol/mL. There is a significant decrease (*p* < 0.01) in MMP1 expression. A more pronounced reduction in MMP1 expression is observed for 160 nmol/mL (***p < 0.001), suggesting a strong effect of M^pro^ on MMP1 expression at high concentrations. Statistically significant differences were observed between the treatment and control (untreated) cells at (**p < 0.01) and (***p < 0.001). To evaluate extracellular MMP1, cell culture media was collected, and an ELISA was performed to assess the release of the membrane‐bound MMP1. Figure 13 illustrates the relative percentage expression of MMP1 in the media of C2C12 cells. In the C2C12 cell line, there is a slight decrease in MMP1 levels for the treatments compared to the control (untreated) cells.

#### 3.2.8 Cellular and Medium DPP4

**Figure 14:**
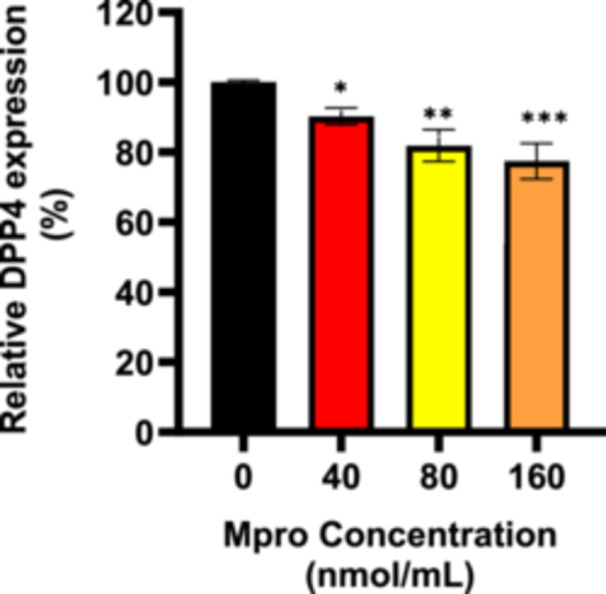
The relative expression of DPP4 was estimated after treating C2C12 and HepG2 cell lines with M^pro^ for 24 h at the different concentrations of 40, 80, and 160 nmol/mL. The values are expressed as mean ± SD represented with error bars (*n* = 3). Results are expressed as a percentage. Untreated cells were used as the control group. The asterisk (*) represents the statistical difference between the test compounds and the control at *p < 0.05, **p < 0.01, ***p < 0.001, and ****p < 0.0001.

**Figure 15:**
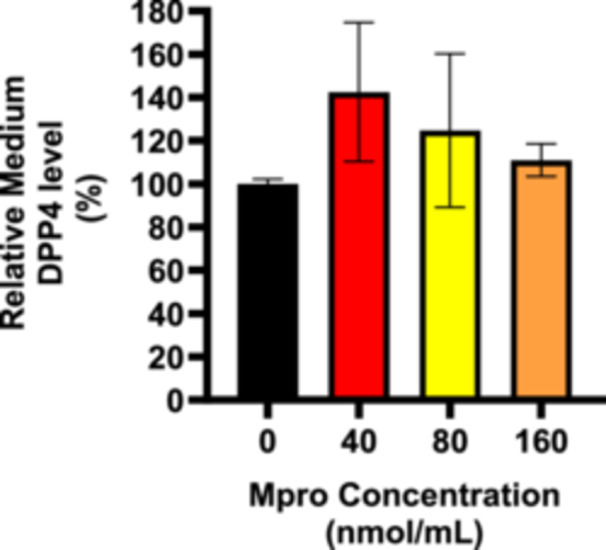
The medium DPP4 was estimated after treating C2C12 and HepG2 cell lines with M^pro^ for 24 h at 40, 80, and 160 nmol/mL concentrations. The values are expressed as mean ± SD represented with error bars (*n* = 3). Untreated cells were used as the control group.

Intracellular DPP4 levels were assessed in the C2C12 cells after 24‐h treatment with M^pro^ at concentrations of 40, 80, and 160 nmol/mL as depicted in Figure 14. There was a decline in DPP4 expression as the concentration of M^pro^ increases by comparison to the control (untreated) cells. Statistically significant differences were observed between all the M^pro^ treatment and control groups at *p < 0.05 for 40 nmol/mL, **p < 0.01 for 80 nmol/mL, and ***p < 0.001 for 160 nmol/mL, revealing a relatively more pronounced effect of M^pro^ with increasing concentrations. To evaluate DPP4 shedding, cell culture media were collected, and an ELISA was performed to assess the release of the membrane‐bound form of DPP4 after treating with M^pro^. Figure 15 illustrates the relative percentage of DPP4 released into cell culture medium in the C2C12 cells. There was a sharp increase in medium DPP4 levels at lower concentrations of M^pro^, followed by a decline at higher concentrations which still above the control group. DPP4 release into the medium increased with the 40 nmol/mL concentration of M^pro^, peaking at this point, although the difference is not statistically significant compared to the control.

#### 3.2.9 Relative MDA Concentration

**Figure 16:**
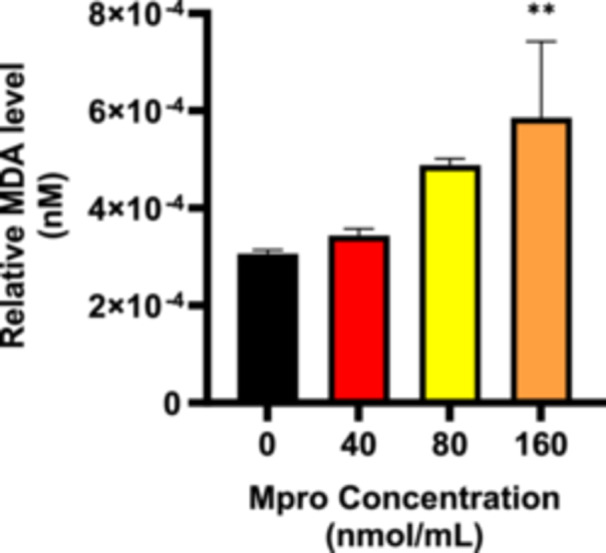
The relative concentration of medium MDA was estimated after treating the C2C12 cell line with M^pro^ for 24 h at the different concentrations of 40, 80, and 160 nmol/mL. The values are expressed as mean ± SD represented with error bars (*n* = 3). Results are expressed as a percentage. Untreated cells were used as the control group. The asterisk (*) represents the statistical difference between the test compounds and the control at **p < 0.01.

Figure 16 depicts the relative percentage medium MDA concentration in the C2C12 cell preparations treated with M^pro^ at concentrations of 40, 80, and 160 nmol/mL, respectively. The MDA levels in the medium increase with an increase in M^pro^ concentration with 40 nmol/mL M^pro^ resulting in a slight, non‐significant increase compared to the control. At 80 nmol/mL, MDA levels rose further, although not statistically significant, compared to the control (untreated) cells. The highest concentration of M^pro^ (160 nmol/mL) led to a statistically significant increase in MDA levels (*p* < 0.01), indicating enhanced oxidative stress at this concentration. This dose‐dependent rise in MDA suggests that M^pro^ treatment induces oxidative stress in C2C12 cells, especially at higher concentrations.

## 4 Discussion

Almost half of the COVID‐19‐related deaths occurred in patients with underlying metabolic and vascular disorders, especially metabolism and endocrine disorders [23]. It has been suggested that COVID‐19 may adversely affect survivors’ endocrine and metabolic systems, including the brain, liver, skeletal muscle, and adipose tissue, which could result in new‐onset hyperglycemia or insulin resistance [23]. As of yet, COVID‐19 remains controversial as to whether it can indirectly or directly lead to new‐onset DM, undiagnosed DM, or prediabetes. The increase in diabetic cases, however, post the COVID‐19 pandemic, has prompted research into the possible mechanistic pathways that the SARS‐CoV‐2 potentially utilizes in inducing DM. Research indicates a link between DM, inflammation, and insulin resistance. This therefore makes it imperative to elucidate the mechanisms that are causing the rapid transition from prediabetes to DM in patients with a history of COVID‐19 infection. Where such insights are shed, necessary steps and strategies could be employed to stop the transition. Furthermore, elucidating the exact mechanisms that the SARS‐CoV‐2 virus is utilizing to cause new‐onset DM will help researchers currently exploring new therapies to target DM in compound selection and target site selection. Therefore, this study aimed to assess the tissue‐specific effects of SARS‐CoV‐2 M^pro^ on glucose handling in vitro. M^pro^ is one of the two SARS‐CoV‐2 enzymes which are involved in the cleaving viral polyproteins process.

From the cytotoxicity study, the C2C12 cell line resisted M^pro^‐induced cytotoxicity, maintaining relatively high viability at low to moderate concentrations. This suggests that C2C12 cells may possess intrinsic protective mechanisms against M^pro^‐induced cytotoxicity, potentially through antioxidant defenses or adaptive stress responses. However, at higher concentrations, a decline in cell viability was observed, indicating that excessive M^pro^ exposure potentially overwhelms these protective mechanisms. Given that skeletal muscle is a major insulin‐sensitive tissue, understanding how M^pro^ affects C2C12 cells could provide insights into its potential role in metabolic dysregulation and post‐COVID‐19 complications, such as insulin resistance or muscle dysfunction.

Glucose transport is widely regarded as the rate‐limiting step in skeletal muscle glucose utilization [24]. Skeletal muscles utilize approximately 80% of postprandial glucose from the circulation [25, 26]. A dose‐dependent inhibition of baseline glucose uptake was evident in C2C12 cells with increasing concentrations of M^pro^. Due to its primary localization on muscle cell surfaces, GLUT1 is considered the key glucose transporter isoform responsible for basal and non‐insulin‐stimulated glucose transport in skeletal muscle tissue [27, 28]. In the study, we suspect that M^pro^ might directly or indirectly affect GLUT1 activity by interacting with GLUT1, altering its structure and function. The lack of GLUT1 quantification is a glaring limitation for this study.

Furthermore, we assessed whether M^pro^ would affect insulin‐stimulated glucose uptake. Insulin physiological action is to enhance glucose disposal via skeletal muscle glucose transport. Therefore, it is imperative to understand the extent of the disturbance in the insulin signaling pathway. M^pro^ was administered in the presence of insulin to investigate this phenomenon. The observations suggest that insulin was unable to effectively mediate glucose uptake as expected, where cells we co‐exposed to M^pro^. As previously stated, the skeletal muscle is capable of reversing whole‐body insulin resistance if the insulin resistance is brought on via skeletal muscles [25]. Therefore, if M^pro^ is inducing an insulin‐resistance state in C2C12 cells, this might be a precipitating factor for the induction of new‐onset DM which led us to further evaluate the effect of M^pro^ on the insulin signaling pathway.

Insulin resistance in skeletal muscle results from reduced activity of early insulin signaling components (IRTK, IRS1, PI3K, and AKT) [28, 29]. Upon activation, the insulin receptor phosphorylates several intracellular substrates, triggering specific signaling pathways. Tyrosine phosphorylation of certain substrates activates PI3K, which generates polyphosphoinositides that interact with protein kinases and ultimately activates the kinase AKT [28, 29]. PI3K/AKT signaling downstream of insulin receptors plays an important role in glucose uptake and glycogen synthesis in skeletal muscles, according to research [30]. Various steps of the insulin signaling pathway are essential for ensuring the correct biological responses to insulin across different tissues. Consequently, disruptions at any point in signal transduction play a significant role in the development of insulin resistance. As previously mentioned, AKT phosphorylation plays a crucial role in the insulin response mediated by PI3K. To explore this possibility, we evaluated AKT expression in the C2C12 cell line.

The reduction in insulin‐stimulated glucose uptake in C2C12 cells could be attributed to various factors, such as M^pro^ interference with the phosphorylation of the downstream effector, AKT in the insulin signaling cascade resulting in impaired translocation of GLUT4 to the plasma membrane or disruption in plasma GLUT4 [31]. In the insulin signaling cascade, AKT phosphorylation leads to GLUT4 storage vesicles (GSVs) release, controlling various stages in the GLUT4 trafficking process such as approaching, tethering, docking, and fusing vesicles with the cell membrane [32]. Different molecules may help coordinate changes in GLUT4 trafficking when insulin is present, but we focused mainly on AKT for our cell‐based assay, as it is the upstream regulator of GLUT4 translocation. Researchers have reported that AKT activation alone, unlike other pathways, is enough to drive GLUT4 translocation to the cell membrane, mimicking insulin's effect [33].

The relative cellular expression of AKT in C2C12 cells showed a dose‐dependent decrease in AKT expression with an increase in M^pro^ concentration. However, the results showed that M^pro^ has a minimum inhibitory effect on baseline AKT expression. Observations of stable baseline levels of AKT allowed us to further explore M^pro^ effects in insulin‐stimulated AKT expression to ascertain the exact disruption in the insulin signaling pathway. Insulin is also a known activator of AKT and the results for insulin‐stimulated AKT expression revealed no significant differences compared to insulin control, indicating that in C2C12 muscle cells, M^pro^ does not seem to significantly impair insulin's ability to activate AKT phosphorylation, even at higher concentrations. These findings suggest that the inhibitory effects in the insulin signaling pathway for C2C12 cells could not therefore be fully attributed to AKT. Our results are not surprising as insulin resistance has been documented even in the absence of reduced phosphorylation of AKT and its activation sites across various models, including insulin‐resistant 3T3‐L1 adipocytes, L6 myotubes, and human muscle both ex vivo and in vivo [34, 35]. Second, research by Jaiswal and colleagues on 3T3‐L1 adipocytes and mouse muscle has shown that only a small portion of the total cellular pool of AKT needs to be active to achieve maximal phosphorylation of its substrates [36]. In other words, if one of the isoforms of AKT is still active, insulin signaling will not be affected. It can bepostulated that changes in signaling molecules beyond AKT in skeletal muscle are required to disrupt glucose tolerance and insulin sensitivity in vivo [37]. Despite the observed interactions between PI3K and M^pro^, the downstream effector AKT showed to be unaffected. Our results indicated that the poor glucose handling could not be attributed to the impairment of PI3K and AKT, leading us to speculate about a disruption at GLUT4 level.

Since the observed poor glucose handling in the C2C12 cell line remains unexplained, this prompted an investigation into the transporters responsible for facilitating glucose uptake. Several studies have demonstrated that the GLUT4 transporter mediates insulin‐stimulated glucose transport in skeletal muscles [38, 39, 40, 41]. Literature suggests that identifying the main factors that cause problems with GLUT4 trafficking could bring significant benefits for treating metabolic diseases. The results for the baseline GLUT4 expression showed that there was no significant difference in GLUT4 between the control (untreated) cells and M^pro^‐treated cells. A plausible explanation for the reduction that was observed in insulin‐stimulated glucose uptake in the face of relatively unaltered GLUT4 content is a reduction in the translocation of GLUT4. This distinction between GLUT4 expression and translocation is critical. While expression refers to the total cellular content of GLUT4, translocation reflects the functional redistribution of GLUT4 to the plasma membrane, where it facilitates glucose uptake. M^pro^ might potentially be inducing an insulin‐resistant state which might be one of the precipitating factors of the new‐onset DM seen in long COVID‐19. As a therapeutic strategy to mitigate SARS‐CoV‐2‐induced insulin resistance, researchers can potentially focus on novel drug designs to develop inhibitors of this interaction as this might help restore GLUT4 function and improve glucose metabolism.

The disruption of insulin signaling was further corroborated by heightened oxidative stress, indicated by increased levels of malondialdehyde (MDA) in the C2C12 cell lines. Lipid peroxidation, which is the reaction between oxygen and unsaturated lipids, generates a diverse range of oxidation products [42]. This process plays a critical role in cellular damage and contributes significantly to oxidative stress in biological systems. The primary products of lipid peroxidation are lipid hydroperoxides. Among the various aldehydes produced as secondary products, MDA is one of the most notable [43]. Lipid peroxidation assays are often used to assess oxidative status in vitro since lipids and lipoproteins are the primary targets of peroxidation in biological membranes. MDA, a key product of lipid oxidation, serves as an important and widely used biomarker for lipid peroxidation [44].

The disturbance in glucose metabolism could also be attributed to the Mpro‐stimulated inflammatory state, as increased by an increase in IL‐6. Inflammation has been reported to be central in the pathogenesis of insulin resistance. Mpro has been reported to elicit inflammation and damaged in the endothelial cells of the brain [45]. Viruses can trigger chronic inflammation, which may lead to cellular transformation [46]. Furthermore, a study by Queiroz and colleagues on cytokine profiles in acute and long‐term COVID‐19 syndrome highlighted IL‐6 as a key cytokine influencing the outcome of COVID‐19, including the disease's duration and severity [47]. Inflammation has also been noted to induce insulin resistance; hence, this could be another potential mechanism in which M^pro^ is inducing glycemic aberrations [48]. The observed increase in both extracellular IL‐6 and medium DPP4 levels with M^pro^ exposure in C2C12 cells suggests a connection between DPP4 and inflammation. Research has shown that there is a well‐established relationship between DPP4 and IL‐6 [49]. In C2C12 cells, M^pro^ gradually lowered intracellular DPP4 expression and increased sDPP4 shedding into the cell medium. Rohrborn et al. shed light on the enzymes involved in DPP4 shedding, identifying that MMP1, MMP2, and MMP14 play a role in regulating this process [50]. Cell culture and rodent models have shown that DPP4 is shed from skeletal muscle membranes through the action of MMPs, but the pathway for release of DPP4 and its function remains unclear [50]. In C2C12 cells, we observed a decrease in both MMP1 and DPP4 intracellular expression, suggesting a potential regulatory relationship. Given that MMP1 is involved in extracellular matrix remodeling and DPP4 plays a role in metabolic regulation, one possibility is that the reduction in both proteins reflects a coordinated response to cellular stress or signaling alterations. M^pro^ might suppress MMP1 production and the consequence of this can be an impairment in the muscle's ability to remodel the ECM effectively resulting in pathophysiology of muscle‐wasting conditions.

In parallel with mechanistic research on M^pro's^ effects on host cells, substantial efforts have been directed toward identifying and developing inhibitors of M^pro^ as antiviral therapeutics [51, 52, 53]. The in silico and experimental studies have reported diverse classes of small molecules and natural products with high predicted affinity for the conserved M^pro^ active site, highlighting its suitability as a drug target due to its central role in viral replication and limited homology with human proteases [51, 52]. In silico investigations have demonstrated that plant‐derived polyphenols, including oxidized Epigallocatechin Gallate (EGCG), can form stable hydrogen‐bond interactions with key catalytic residues of M^pro^, potentially blocking its activity and modulating inflammatory pathways [51]. Broader virtual screening and structural analyses in related work have similarly identified multiple scaffolds capable of occupying conserved M^pro^ binding pockets. Natural products such as cordifolioside, berberine, and magnoflorine have also been predicted through molecular docking to inhibit M^pro^ by occupying its conserved binding pockets [52]. Collectively, these studies underscore M^pro^ as a key antiviral target and support ongoing efforts to screen both natural and synthetic compounds for inhibitory potential, which may also intersect with host metabolic and inflammatory pathways implicated in COVID‐19‐related insulin resistance.

While this study was conducted in vitro to elucidate mechanistic insights, the findings carry potential clinical relevance. The observation that M^pro^ potentially disrupts glucose handling in skeletal muscle cells suggests a molecular mechanism that could contribute to the dysglycemia and new‐onset diabetes reported in some COVID‐19 patients. For clinicians, these results underscore the importance of monitoring glucose metabolism in patients recovering from SARS‐CoV‐2 infection, even in those without a prior history of diabetes and may encourage clinicians to consider early screening and intervention for glucose abnormalities. Patients with prediabetes may be at a higher risk of converting to full diabetes with SARS‐Cov‐2 infection, which can therefore increase diabetes prevalence. For these reasons, epidemiological studies aiming at uncovering diabetes prevalence post‐COVID‐19 pandemic should be accelerated. Most importantly, clinicians and public health authorities should consider adopting a history of SARS‐Cov‐2 infection as a risk factor for developing diabetes. For Type 1 diabetes, SARS‐CoV‐2 infection may result into insulin resistance in part through the mechanism our study has demonstrated. This can therefore necessitate a higher dose of insulin or perhaps inclusion of insulin sensitizers for patients to overcome insulin resistance. Indeed, recent clinical studies have found that there are increased insulin requirements in severe cases of Covid‐19 are higher than in moderate cases [54, 55, 56]. For such patients, constant medication review may be necessary.

To extend these insights, future in vivo studies are needed to confirm the physiological relevance of our findings. Murine models of diet‐induced insulin resistance or systems engineered to express SARS‐CoV‐2 proteins could help determine whether M^pro^ impairs glucose handling at the whole‐organism level. Such approaches would allow evaluation of systemic outcomes, including glucose tolerance, insulin sensitivity, and tissue‐specific signaling, thereby bridging the gap between cellular mechanisms and clinical observations.

While our study provides novel insights into the potential effects of SARS‐CoV‐2 M^pro^ on skeletal muscle cells, several limitations should be acknowledged. The work was conducted in vitro using the C2C12 cell line, which may not fully capture the physiological complexity of human tissues, especially pancreatic β‐cells and adipocytes. Furthermore, while this study explored the disturbances in glucose uptake and GLUT4 translocation, it did not explore upstream insulin signaling components such as IRS1or PI3K. Our work only lays the foundation for future studies to map the broader signaling cascade and clarify the precise molecular mechanisms through which M^pro^ disrupts glucose metabolism. Finally, the absence of in vivo studies or patient‐derived samples restricts direct clinical translation, and given the multifactorial nature of diabetes, M^pro's^ role should be considered as one of several possible contributors to new‐onset metabolic dysfunction post‐COVID‐19.

Taken together, our findings highlight the intricate interplay between DM and SARS‐CoV‐2, warranting further research to elucidate these pathways and their broader physiological and pathological impact on new‐onset DM. Taken together, our study could demonstrate that SARS‐CoV‐2 M^pro^ presents itself as a risk factor for insulin resistance development and T2DM. The potential mechanisms we have illuminated in our findings should further consolidate evidence linking COVID‐19 with the onset of DM.

## 5 Conclusion

The global rise in DM was anticipated even before the emergence of COVID‐19‐induced diabetes. However, the pandemic has accelerated our understanding of DM's pathogenesis, particularly the role of viral infections in metabolic reprogramming and glycemic dysregulation. Understanding the cellular and molecular mechanisms of glucose metabolism is crucial for establishing links between these diseases and developing targeted therapeutic interventions. This study highlights the potential impact of COVID‐19 M^pro^ on glucose metabolism, suggesting that it disrupts insulin signaling in skeletal muscles. Our findings reveal impaired glucose uptake, reduced GLUT4 translocation and alterations in the AKT pathway which are factors that may contribute to insulin resistance, a key driver of DM and metabolic disorders. Additionally, elevated IL‐6 levels suggest that systemic inflammation plays a role in these metabolic disturbances. While current evidence is insufficient to conclusively link M^pro^ to hyperglycemia in long‐COVID‐19 patients, this study indicates that M^pro^ interferes with insulin signaling. Given the observed disruptions in glucose handling, GLUT4 function, and inflammatory markers, M^pro^ effect appears to be primarily indirect, likely involving intracellular signaling proteins rather than direct insulin interaction. Despite significant progress, many gaps remain in understanding the molecular mechanisms underlying new‐onset DM post‐COVID‐19. Further research is needed to validate these in vitro findings and explore potential therapeutic strategies to mitigate these effects in COVID‐19 patients.

## Author Contributions

**Praise Tatenda Nhau:** conceptualization, investigation, writing – original draft. **Mlindeli Gamede:** supervision, writing – review and editing, validation. **Andile Khathi:** supervision, writing – review and editing, validation. **Ntethelelo Sibiya:** supervision, writing – review and editing, validation.

## Ethics Statement

The authors have nothing to report.

## Consent

The authors have nothing to report.

## Conflicts of Interest

The authors declare no conflicts of interest.

## Data Availability

Supporting data are available upon request.

## References

[1] M. Nassar , A. Daoud , N. Nso , et al., “Diabetes Mellitus and COVID‐19,” Diabetes & Metabolic Syndrome: Clinical Research & Reviews 15, no. 6 (2021): 102268, 10.1016/j.dsx.2021.102268. PMC8416292 34562865

[2] D. O. Abegunde , C. D. Mathers , T. Adam , M. Ortegon , and K. Strong , “The Burden and Costs of Chronic Diseases in Low‐Income and Middle‐Income Countries,” Lancet 370, no. 9603 (December 2007): 1929–1938, 10.1016/S0140-6736(07)61696-1. 18063029

[3] K. Kiernan and N. J. MacIver , “Viral Infection “Interferes” With Glucose Tolerance,” Immunity 49, no. 1 (July 2018): 6–8, 10.1016/j.immuni.2018.06.013. 30021147

[4] S. U. Jeong , D. G. Kang , D. H. Lee , et al., “Clinical Characteristics of Type 2 Diabetes Patients According to Family History of Diabetes,” Korean Diabetes Journal 34, no. 4 (August 2010): 222–228, 10.4093/kdj.2010.34.4.222. 20835339 PMC2932891

[5] R. D. Leslie , J. Palmer , N. C. Schloot , and A. Lernmark , “Diabetes at the Crossroads: Relevance of Disease Classification to Pathophysiology and Treatment,” Diabetologia 59, no. 1 (January 2016): 13–20, 10.1007/s00125-015-3789-z. 26498592

[6] D. O. Abegunde , C. D. Mathers , T. Adam , M. Ortegon , and K. Strong , “The Burden and Costs of Chronic Diseases in Low‐Income and Middle‐Income Countries,” Lancet 370, no. 9603 (December 2007): 1929–1938, 10.1016/S0140-6736(07)61696-1. 18063029

[7] A. K. Khetan and S. Rajagopalan , “Prediabetes,” Canadian Journal of Cardiology 34, no. 5 (May 2018): 615–623, 10.1016/j.cjca.2017.12.030. 29731022

[8] W. Sami , T. Ansari , N. S. Butt , and M. R. A. Hamid , “Effect of Diet on Type 2 Diabetes Mellitus: A Review,” International Journal of Health Sciences 11, no. 2 (2017): 65–71. PMC5426415 28539866

[9] M. A. Abdul‐Ghani and R. A. DeFronzo , “Pathophysiology of Prediabetes,” Current Diabetes Reports 9, no. 3 (June 2009): 193–199, 10.1007/s11892-009-0037-y. 19490820

[10] N. Sibiya , N. Mzimela , B. Mbatha , P. Ngubane , and A. Khathi , “The Insights on Why Diabetes Prevalence May Increase Amid or Post COVID‐19 Pandemic,” Current Diabetes Reviews 19, no. 4 (January 2023): 37–45, 10.2174/1573399818666220411122345. 35410612

[11] M. Šestan , S. Marinović , I. Kavazović , et al., “Virus‐Induced Interferon‐γ Causes Insulin Resistance in Skeletal Muscle and Derails Glycemic Control in Obesity,” Immunity 49, no. 1 (July 2018): 164–177.e6, 10.1016/j.immuni.2018.06.017. 29958802

[12] A. Coppelli , R. Giannarelli , M. Aragona , et al., “Hyperglycemia at Hospital Admission Is Associated With Severity of the Prognosis in Patients Hospitalized for COVID‐19: The Pisa COVID‐19 Study,” Diabetes Care 43, no. 10 (August 2020): 2345–2348, 10.2337/dc20-1380. 32788285

[13] G. Iacobellis , C. A. Penaherrera , L. E. Bermudez , and E. Bernal Mizrachi , “Admission Hyperglycemia and Radiological Findings of SARS‐CoV2 in Patients With and Without Diabetes,” Diabetes Research and Clinical Practice 164 (June 2020): 108185, 10.1016/j.diabres.2020.108185. 32360710 PMC7251996

[14] O. A. Parlițeanu , M. A. Bălteanu , C. Zaharia , et al., “The Impact of SARS‐CoV‐2 Infection on Glucose Homeostasis in Hospitalized Patients With Pulmonary Impairment,” Diagnostics 15, no. 5 (February 2025): 554. 40075801 10.3390/diagnostics15050554 PMC11898410

[15] A. A. Metwally , P. Mehta , B. S. Johnson , A. Nagarjuna , and M. P. Snyder , “COVID‐19‐Induced New‐Onset Diabetes: Trends and Technologies,” Diabetes 70, no. 12 (2021): 2733–2744, 10.2337/dbi21-0029. 34686519 PMC8660988

[16] R. Pal and M. Banerjee , “COVID‐19 and the Endocrine System: Exploring the Unexplored,” Journal of Endocrinological Investigation 43, no. 7 (July 2020): 1027–1031, 10.1007/s40618-020-01276-8. 32361826 PMC7195612

[17] B. J. Biesiadecki and J. P. Jin , “A High‐Throughput Solid‐Phase Microplate Protein‐Binding Assay to Investigate Interactions Between Myofilament Proteins,” Journal of Biomedicine & Biotechnology 2011 (2011): 421701, 10.1155/2011/421701. 22190850 PMC3228687

[18] S. Sharma and S. Deep , “In‐Silico Drug Repurposing for Targeting SARS‐CoV‐2 Main Protease (Mpro),” Journal of Biomolecular Structure and Dynamics 40, no. 7 (May 2022): 3003–3010. 33179568 10.1080/07391102.2020.1844058 PMC7678360

[19] Q. Hu , Y. Xiong , G. H. Zhu , et al., “The SARS‐CoV‐2 Main Protease (Mpro): Structure, Function, and Emerging Therapies for COVID‐19,” MedComm 3, no. 3 (September 2022): e151. 35845352 10.1002/mco2.151 PMC9283855

[20] H. Su , S. Yao , W. Zhao , et al., “Identification of Pyrogallol as a Warhead in Design of Covalent Inhibitors for the SARS‐CoV‐2 3CL Protease,” Nature Communications 12, no. 1 (2021): 3623. 10.1038/s41467-021-23751-3 PMC8206078 34131140

[21] J. C. Ferreira , S. Fadl , A. J. Villanueva , and W. M. Rabeh , “Catalytic Dyad Residues His41 and Cys145 Impact the Catalytic Activity and Overall Conformational Fold of the Main SARSCoV‐2 Protease 3‐Chymotrypsin‐Like Protease,” Frontiers in Chemistry 9 (2021): e692168, https://www.frontiersin.org/articles/10.3389/fchem.2021.692168. 10.3389/fchem.2021.692168 PMC8264439 34249864

[22] A. Kuretu , M. Mothibe , P. Ngubane , and N. Sibiya , “Elucidating the Effect of Drug‐Induced Mitochondrial Dysfunction on Insulin Signaling and Glucose Handling in Skeletal Muscle Cell Line (C2C12) In Vitro,” PLoS One 19, no. 9 (2024): e0310406, 10.1371/journal.pone.0310406. 39288128 PMC11407670

[23] C. Steenblock , P. E. H. Schwarz , B. Ludwig , et al., “COVID‐19 and Metabolic Disease: Mechanisms and Clinical Management,” Lancet Diabetes & Endocrinology 9, no. 11 (November 2021): 786–798, 10.1016/S2213-8587(21)00244-8. 34619105 PMC8489878

[24] K. E. Merz and D. C. Thurmond , “Role of Skeletal Muscle in Insulin Resistance and Glucose Uptake,” Comprehensive Physiology 10 (2020): 785–809, 10.1002/cphy.c190029. 32940941 PMC8074531

[25] A. Chadt and H. Al‐Hasani , “Glucose Transporters in Adipose Tissue, Liver, and Skeletal Muscle in Metabolic Health and Disease,” Pflügers Archiv ‐ European Journal of Physiology 472, no. 9 (September 2020): 1273–1298, 10.1007/s00424-020-02442-8. 32591906 PMC7462924

[26] C. Y. Wong , H. Al‐Salami , and C. R. Dass , “C2C12 Cell Model: Its Role in Understanding of Insulin Resistance at the Molecular Level and Pharmaceutical Development at the Preclinical Stage,” Journal of Pharmacy and Pharmacology 72, no. 12 (December 2020): 1667–1693, 10.1111/jphp.13300. 32812252

[27] X. X. Han and A. Bonen , “Epinephrine Translocates GLUT‐4 but Inhibits Insulin‐Stimulated Glucose Transport in Rat Muscle,” American Journal of Physiology‐Endocrinology and Metabolism 274, no. 4 (April 1998): E700–E707, 10.1152/ajpendo.1998.274.4.E700. 9575832

[28] A. Marette , J. M. Richardson , T. Ramlal , et al., “Abundance, Localization, and Insulin‐Induced Translocation of Glucose Transporters in Red and White Muscle,” American Journal of Physiology‐Cell Physiology 263, no. 2 (August 1992): C443–C452, 10.1152/ajpcell.1992.263.2.C443. 1514590

[29] S. H. Lee , S. Y. Park , and C. S. Choi , “Insulin Resistance: From Mechanisms to Therapeutic Strategies,” Diabetes & Metabolism Journal 46, no. 1 (January 2022): 15–37, 10.4093/dmj.2021.0213. 34965646 PMC8831809

[30] S. Fröjdö , H. Vidal , and L. Pirola , “Alterations of Insulin Signaling in Type 2 Diabetes: A Review of the Current Evidence From Humans,” Biochimica et Biophysica Acta (BBA) ‐ Molecular Basis of Disease Molecular Basis of Disease 1792, no. 2 (February 2009): 83–92, 10.1016/j.bbadis.2008.10.006. 19041393

[31] M. Li , X. Chi , Y. Wang , S. Setrerrahmane , W. Xie , and H. Xu , “Trends in Insulin Resistance: Insights into Mechanisms and Therapeutic Strategy,” Signal Transduction and Targeted Therapy 7, no. 1 (July 2022): 216, 10.1038/s41392-022-01039-8. 35794109 PMC9259665

[32] A. R. Saltiel , “Insulin Signaling in Health and Disease,” Journal of Clinical Investigation 131, no. 1 (January 2021): e142241, 10.1172/JCI142241. 33393497 PMC7773347

[33] J. Stöckli , D. J. Fazakerley , and D. E. James , “GLUT4 Exocytosis,” Journal of Cell Science 124, no. 24 (December 2011): 4147–4159, 10.1242/jcs.097337. 22247191 PMC3258103

[34] J. Van Gerwen , A. S. Shun‐Shion , and D. J. Fazakerley , “Insulin Signalling and GLUT4 Trafficking in Insulin Resistance,” Biochemical Society Transactions 51, no. 3 (May 2023): 1057–1069, 10.1042/BST20221066. 37248992 PMC10317183

[35] P. A. Ramos , K. A. Lytle , D. Delivanis , S. Nielsen , N. K. LeBrasseur , and M. D. Jensen , “Insulin‐Stimulated Muscle Glucose Uptake and Insulin Signaling in Lean and Obese Humans,” Journal of Clinical Endocrinology and Metabolism 106, no. 4 (April 2021): 1631–1646, 10.1210/clinem/dgaa967. PMC7993573 33382888

[36] N. Jaiswal , M. G. Gavin , W. J. Quinn , et al., “The Role of Skeletal Muscle Akt in the Regulation of Muscle Mass and Glucose Homeostasis,” Molecular Metabolism 28 (October 2019): 1–13, 10.1016/j.molmet.2019.07.004. 31444134 PMC6822261

[37] D. J. Fazakerley , A. Y. Minard , J. R. Krycer , et al., “Mitochondrial Oxidative Stress Causes Insulin Resistance Without Disrupting Oxidative Phosphorylation,” Journal of Biological Chemistry 293, no. 19 (May 2018): 7315–7328, 10.1074/jbc.RA117.001549. 29599292 PMC5950018

[38] D. E. James , R. Brown , J. Navarro , and P. F. Pilch , “Insulin‐Regulatable Tissues Express a Unique Insulin‐Sensitive Glucose Transport Protein,” Nature 333, no. 6169 (May 1988): 183–185, 10.1038/333183a0. 3285221

[39] A. Zorzano , L. Sevilla , E. Tomàs , M. Camps , A. Gumà , and M. Palacín , “Trafficking Pathway of GLUT4 Glucose Transporters in Muscle (Review),” International Journal of Molecular Medicine 2, no. 3 (September 1998): 263–271, 10.3892/ijmm.2.3.263. 9855697

[40] S. Rea and D. E. James , “Moving GLUT4: the Biogenesis and Trafficking of GLUT4 Storage Vesicles,” Diabetes 46, no. 11 (November 1997): 1667–1677, 10.2337/diab.46.11.1667. 9356011

[41] P. L. Evans , S. L. McMillin , L. A. Weyrauch , and C. A. Witczak , “Regulation of Skeletal Muscle Glucose Transport and Glucose Metabolism by Exercise Training,” Nutrients 11, no. 10 (October 2019): 2432, 10.3390/nu11102432. 31614762 PMC6835691

[42] A. Ayala , M. F. Muñoz , and S. Argüelles , “Lipid Peroxidation: Production, Metabolism, and Signaling Mechanisms of Malondialdehyde and 4‐Hydroxy‐2‐Nonenal,” Oxidative Medicine and Cellular Longevity 2014;2014 (2014): 360438, 10.1155/2014/360438. 24999379 PMC4066722

[43] H. Esterbauer , P. Eckl , and A. Ortner , “Possible Mutagens Derived From Lipids and Lipid Precursors,” Mutation Research/Reviews in Genetic Toxicology 238, no. 3 (May 1990): 223–233, 10.1016/0165-1110(90)90014-3. 2342513

[44] F. Ito , Y. Sono , and T. Ito , “Measurement and Clinical Significance of Lipid Peroxidation as a Biomarker of Oxidative Stress: Oxidative Stress in Diabetes, Atherosclerosis, and Chronic Inflammation,” Antioxidants 8, no. 3 (March 2019): 72, 10.3390/antiox8030072. 30934586 PMC6466575

[45] J. Wenzel , J. Lampe , H. Müller‐Fielitz , et al., “The SARS‐CoV‐2 Main Protease Mpro Causes Microvascular Brain Pathology by Cleaving NEMO in Brain Endothelial Cells,” Nature Neuroscience 24, no. 11 (November 2021): 1522–1533, 10.1038/s41593-021-00926-1. 34675436 PMC8553622

[46] M. Z. Tay , C. M. Poh , L. Rénia , P. A. MacAry , and L. F. P. Ng , “The Trinity of COVID‐19: Immunity, Inflammation and Intervention,” Nature Reviews Immunology 20, no. 6 (June 2020): 363–374, 10.1038/s41577-020-0311-8. PMC7187672 32346093

[47] M. A. F. Queiroz , P. F. M. Neves , S. S. Lima , et al., “Cytokine Profiles Associated With Acute COVID‐19 and Long COVID‐19 Syndrome,” Frontiers in Cellular and Infection Microbiology 12 (2022): 922422, 10.3389/fcimb.2022.922422. 35846757 PMC9279918

[48] P. T. Nhau , M. Gamede , and N. Sibiya , “COVID‐19‐Induced Diabetes Mellitus: Comprehensive Cellular and Molecular Mechanistic Insights,” Pathophysiology 31, no. 2 (April 2024): 197–209, 10.3390/pathophysiology31020016. 38651404 PMC11036300

[49] N. Wronkowitz , S. W. Görgens , T. Romacho , et al., “Soluble DPP4 Induces Inflammation and Proliferation of Human Smooth Muscle Cells via Protease‐Activated Receptor 2,” Biochimica et Biophysica Acta (BBA) ‐ Molecular Basis of Disease 1842, no. 9 (September 2014): 1613–1621, 10.1016/j.bbadis.2014.06.004. 24928308

[50] D. Röhrborn , J. Eckel , and H. Sell , “Shedding of Dipeptidyl Peptidase 4 Is Mediated by Metalloproteases and Up‐Regulated by Hypoxia in Human Adipocytes and Smooth Muscle Cells,” FEBS Letters 588, no. 21 (November 2014): 3870–3877, 10.1016/j.febslet.2014.08.029. 25217834

[51] R. Ungarala , M. Munikumar , S. N. Sinha , D. Kumar , R. S. Sunder , and S. Challa , “Assessment of Antioxidant, Immunomodulatory Activity of Oxidised Epigallocatechin‐3‐Gallate (Green Tea Polyphenol) and Its Action on the Main Protease of SARS‐CoV‐2—An In Vitro and In Silico Approach,” Antioxidants 11, no. 2 (2022): 294, 10.3390/antiox11020294. 35204178 PMC8868081

[52] T. S. Ram , M. Munikumar , V. N. Raju , et al., “In Silico Evaluation of the Compounds of the Ayurvedic Drug, AYUSH‐64, for the Action Against the SARS‐CoV‐2 Main Protease,” Journal of Ayurveda and Integrative Medicine 13, no. 1 (January 2022): 100413, 10.1016/j.jaim.2021.02.004. 33654345 PMC7906523

[53] M. Manne , G. Goudar , S. R. Varikasuvu , et al., “Cordifolioside: Potent Inhibitor Against M^pro^ of SARS‐CoV‐2 and Immunomodulatory Through Human TGF‐β and TNF‐α,” 3 Biotech 11 (2021): 136, 10.1007/s13205-021-02685-z. PMC7898013 33643762

[54] T. Matsui , E. Ushigome , M. Hamaguchi , et al., “Increased Insulin Requirements in Severe Cases of Covid‐19 Are Higher Than in Moderate Cases,” Diabetes, Metabolic Syndrome and Obesity 17 (October 2024): 3727–3733, 10.2147/DMSO.S480598. PMC11558443 39539455

[55] N. Soltani , H. Häbel , A. Balintescu , et al., “Insulin Requirement Trajectories During COVID‐19 Versus non‐COVID‐19 Critical Illness—A Retrospective Cohort Study,” Acta Anaesthesiologica Scandinavica 69, no. 1 (January 2025): e14536. 39402855 10.1111/aas.14536

[56] B. Del Carpio , J. Trang , M. Vallejo , et al., “High‐Dose Insulin Infusion in Patients With COVID‐19,” BMJ Open Diabetes Research & Care 9, no. 2 (November 2021): e002415. 10.1136/bmjdrc-2021-002415 PMC8587530 34764139

